# Charting the growth of idiopathic short stature research: a 32-year bibliometric study of global advances

**DOI:** 10.3389/fendo.2025.1667011

**Published:** 2025-11-19

**Authors:** Zhao Wang, Zhendong Gao, Yongdie Huang, Zhen Li, Fei Luo, Tao Li, Yu Zhang, Lili Lin, Shouchuan Wang, Jin Ye

**Affiliations:** 1 Department of Pediatrics, Affiliated Hospital of Nanjing University of Chinese Medicine, Nanjing, China; 2 Jiangsu Key Laboratory of Children’s Health and Chinese Medicine, Institute of Pediatrics, Medical Metabolomics Center, Nanjing, China; 3 Jiangsu Provincial Research Institute of Chinese Medicine Schools, Jiangsu, China; 4 Department of Thoracic Surgery and State Key Laboratory of Genetics and Development of Complex Phenotypes, Fudan University Shanghai Cancer Center, Shanghai, China; 5 Institute of Thoracic Oncology, Fudan University, Shanghai, China; 6 Department of Oncology, Shanghai Medical College, Fudan University Shanghai, Shanghai, China; 7 Jinan Shizhong District Ganshiqiao Sub-district Office Jingqi Road Community Health Service Station, Jinan, China; 8 Jiangyin Affiliated Hospital of Nanjing University of Traditional Chinese Medicine, Jiangyin, China

**Keywords:** bibliometric analysis, endocrinology, growth hormone, idiopathic short stature, precision medicine

## Abstract

**Introduction:**

Idiopathic short stature (ISS) is a common paediatric endocrinological disorder defined by a height > two standard deviations (SDs) below the mean for age, sex, and ethnicity without an identifiable cause. This condition accounts for 60–80% of short stature cases in children and presents with complex and heterogeneous aetiology. ISS-related research has expanded rapidly over the past three decades; however, a comprehensive bibliometric analysis outlining its developmental trajectory and identifying emerging research priorities remains unavailable. Therefore, using network visualisation tools, we mapped 32 years of research to identify key trends, influential contributors, relevant journals, and emerging directions in ISS.

**Methods:**

Publications related to ISS in children, published between January 1993 and December 2024, were retrieved from the Web of Science Core Collection. Bibliometric and visual analyses were conducted using CiteSpace 6.1.R6, VOSviewer 1.6.20 and R package Bibliometrix. The analysis examined multiple dimensions, including annual publication trends, country and institutional contributions, collaboration networks, influential authors and journals, keyword evolution, and co-cited references to map the global ISS research landscape. We retrieved clinical trials published between 1993 and 2024 from the PubMed database to analyze the clinical progress in this field.

**Results:**

A total of 1, 567 articles were included in the analysis. The volume of publications in the ISS field has steadily increased over time. The United States of America contributed the most publications. Leiden University in the Netherlands produced the highest number of institutional publications. Wit authored the greatest number of publications, whereas Ranke had the highest number of citations. *The Journal of Clinical Endocrinology & Metabolism* ranked first in both publication count and citation frequency. Co-citation analysis identified foundational documents, including the 2008 expert consensus led by Cohen, and the 2016 clinical guidelines spearheaded by Grimberg. Recent keyword bursts such as “heterozygous mutation, “ “guideline, “ and “variant” suggest growing attention to genetic mechanisms and precision medicine.

**Discussion:**

This study systematically mapped the developmental trajectory and thematic evolution of ISS research over the past three decades using a comprehensive bibliometric analysis. The findings identified key contributors and research hotspots, providing valuable insights and reference points to guide future investigations.

## 1 Introduction

Idiopathic short stature (ISS) refers to a heterogeneous group of growth disorders with an undefined aetiology, defined as a height more than two standard deviations (SDs) below the mean for age, sex, and ethnicity. Children diagnosed with ISS typically present with normal birth length, weight, and body proportions. Clinical evaluation reveals no evidence of systemic, endocrine, nutritional, chromosomal, or genetic abnormalities (1). Diagnosing ISS requires the exclusion of identifiable causes and presents certain clinical challenges. Epidemiological studies have found that approximately 60–80% of children with a height ≤ -2 SDs meet the criteria for ISS (2). The underlying mechanisms of ISS are multifactorial and involve dysregulation of the growth hormone–insulin-like growth factor (GH–IGF) axis, dysfunction of growth plate chondrocytes, and alterations in genetic regulatory networks (3–6).

Clinically, ISS reduces final adult height, impairs psychosocial well-being, and increases long-term cardiovascular risk (7, 8). Currently, recombinant human growth hormone (rhGH) is the only United States of America’s (USA’s) Food and Drug Administration (FDA)-approved treatment for children with ISS (9). Although rhGH has been shown to improve final height in many patients, treatment responses vary markedly. Adverse events include increased intracranial pressure, slipped capital femoral epiphysis, and scoliosis, which require long-term safety monitoring (10–12). Despite these advancements in understanding and treatment, there remains a notable absence of comprehensive macroscopic analyses detailing the evolution of ISS research. Although the existing literature is rich in specific clinical and molecular studies, it has not systematically mapped the intellectual landscape of the field. ISS-related research has steadily increased over the past three decades; however, no systematic analysis of the field’s development and current landscape exists.

Bibliometrics, an interdisciplinary method that integrates statistics and informatics, allows for the quantitative analysis of scientific literature. By examining the distribution of nations, institutions, authors, keywords, and references, bibliometric analysis tracks academic development, identifies research hotspots, and assesses scholarly influence (13–15). The Web of Science (WOS), a comprehensive academic database, includes high-impact journals in multiple disciplines. Therefore, the primary limitation of this study is the absence of a holistic, quantitative overview of research trends and the intellectual structure within the field of ISS. In this study, we conducted a bibliometric analysis of ISS-related publications indexed in the WOS from 1 January, 1993 to 31 December, 2024. Using network visualisation tools, we systematically reviewed the past 32 years of ISS research, highlighting key developmental trends and current research dynamics. Our objective was to guide researchers, particularly new entrants to the field, by mapping influential authors and references, identifying suitable journals, and highlighting emerging directions in ISS research. This study presents the first comprehensive bibliometric mapping of the ISS research landscape, offering new insights into its growth, collaborative networks, thematic shifts, and impact over more than three decades.

## 2 Methods

Compared with Google Scholar and Scopus, the Web of Science Core Collection (WoSCC) provides higher-quality journal indexing and more comprehensive citation data (16, 17). Concurrently, PubMed provides extensive coverage of biomedical and clinical research literatures with specialized precision filtering for publication types. This combined infrastructure enables efficient and accurate retrieval of clinical trial progress related to ISS. Therefore, we conducted comprehensive searches of WoSCC and PubMed for publications dated 1 January 1993 to 31 December 2024. Only English-language records were included.

For WoSCC, we used the Topic field (TS, including title, abstract, author keywords, and Keywords Plus), with limits applied for document type, language, and year. The exact Advanced Search query was TS = (“idiopathic short stature” OR “idiopathic dwarfism” OR “idiopathic dwarf”) AND DT = (Article OR Review) AND LA=(English) AND PY = (1993-2024). This search retrieved 1, 567 records, which were exported with cited references in plain-text format for analysis. For clinical progress assessment, PubMed was searched using the Title/Abstract field tag with publication-type, language, and date restrictions. The exact query was: (“idiopathic short stature”[Title/Abstract] OR “idiopathic dwarfism”[Title/Abstract] OR “idiopathic dwarf”[Title/Abstract]) AND (Clinical Trial [Publication Type] OR Randomized Controlled Trial [Publication Type]) AND (“1993/01/01”[Date - Publication]: “2024/12/31”[Date - Publication]) AND English [Language]. Data cleaning and deduplication were conducted through a two-phase protocol. In Phase 1, we retained only Article and Review publications (DT field) while excluding non-research publication types (Editorials, Letters, Meeting Abstracts, etc.). Language (LA = English) and publication year (PY = 1993-2024) filters were systematically applied. In Phase 2, duplicate removal via composite matching of DOI, title, and author-publication year combinations; geopolitical unification adhering to ISO 3166–1 standards (e.g., “Peoples R China” and “Taiwan” were grouped under “China”; “United States of America” was standardized as “USA”; and “England, “ “Scotland, “ “Wales, “ and “Northern Ireland” were merged into “UK.”).

To reduce lexical fragmentation, synonym/thesaurus files in CiteSpace 6.1.R6, VOSviewer 1.6.20, and the bibliometrix R package (18, 19) were used to merge duplicate or variant terms (e.g., “GH” and “growth hormone”), correct spelling inconsistencies, and remove non-informative keywords. Reference metadata were imported into these tools to identify core countries, institutions, authors, journals, keywords, and influential references. Title and abstract screening were conducted independently by two reviewers; disagreements were resolved through discussion, and unresolved conflicts were adjudicated by a third reviewer.

## 3 Results

### 3.1 Annual publication trends

**Figure 1:**
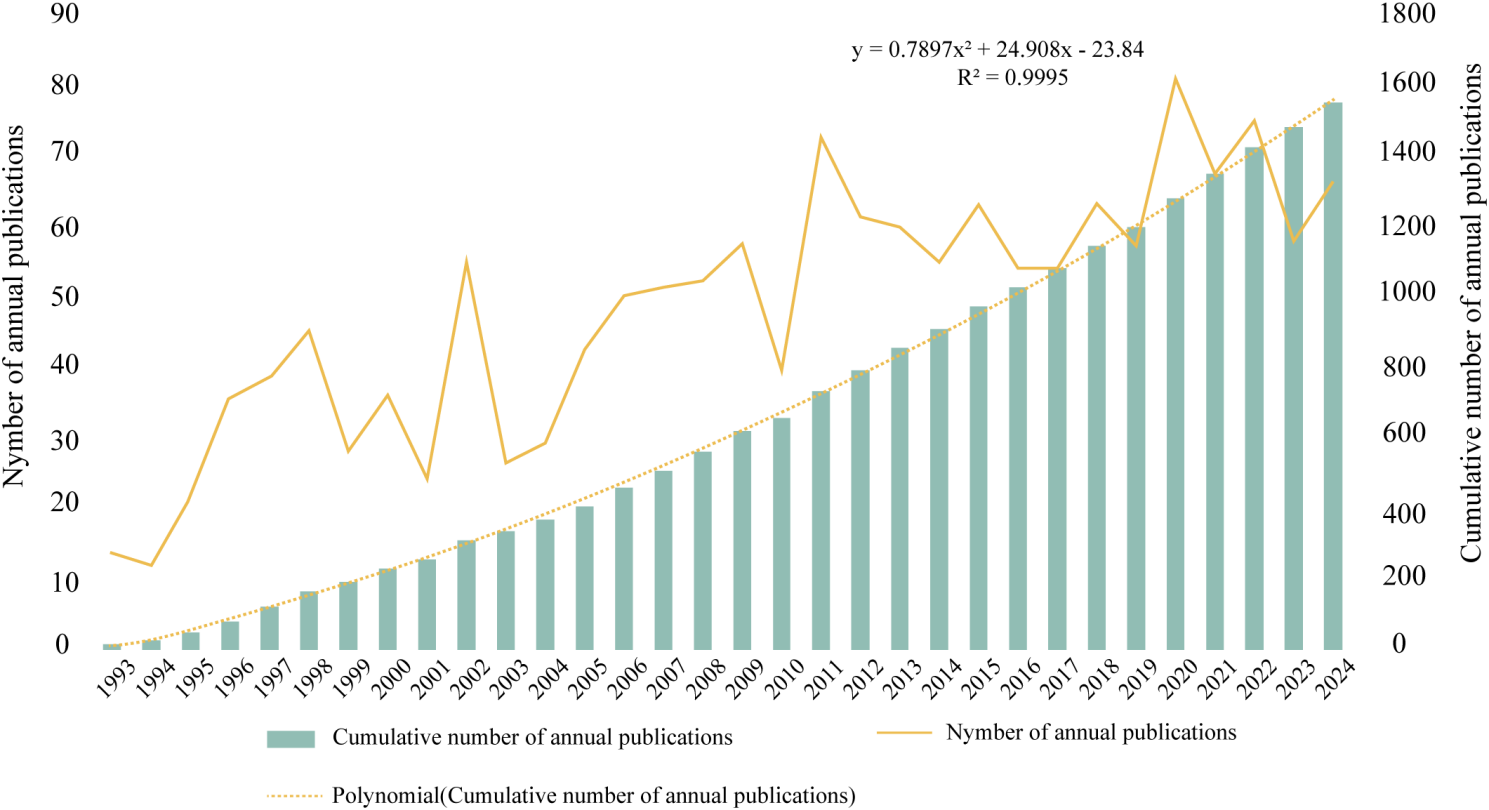
Annual and cumulative publication trends in the field of ISS from 1993 to 2024 The bar graph presents the cumulative number of publications per year, and the yellow line indicates the number of annual publications. A second-order polynomial model (y= 0.7897x² + 24.908x -23.84; R²= 0.9995) fits the cumulative growth curve and reflects a steady, accelerating increase in research output over the past three decades.

From 1993 to 2024, 1, 567 ISS-related publications were indexed in the WoSCC database, comprising 1, 344 original research articles and 223 reviews. As shown in **Figure 1**, the annual publication count demonstrated an overall upward trajectory with periodic fluctuations. During 1993–1996, publication volume remained relatively low at approximately 10–30 per year. A notable increase was observed between 1997 and 2002, particularly between 2001 and 2002. Between 2003 and 2009, output stabilised between 30 and 60 articles annually. After 2010, steady growth resumed, peaking in 2020, with > 80 publications. From 2021 to 2023, output ranged from 60 to 80 annually, reflecting sustained research activity. A polynomial regression model of cumulative publication volume demonstrated a strong fit (R² = 0.9995), with a quadratic growth trend described:  y= 0.7897x² + 24.908x − 23.84, indicating continued growth in ISS literature. Overall, ISS has emerged as a key research focus in paediatric endocrinology, growth, and development; and the field remains in an active phase of expansion.

### 3.2 Country and institutional analysis

**Tables 1**, **2** present the top five nations and institutions based on publication volume. The USA ranked first in ISS-related publications (n= 465; 20.97% of the total), followed by Germany and the United Kingdom (UK). At the institutional level, Leiden University in the Netherlands held the highest output (n= 60; 1.91% of the total), followed by the University of Gothenburg in Sweden and Oregon Health & Science University in the USA. **Figures 2A, C** illustrate the collaborative networks among nations involved in ISS research. Node size reflects the number of publications from each country, and connecting lines represent cooperative relationships. Nations with purple outer rings exhibit high betweenness centrality. The visualised network shows that the USA holds a central position, maintaining extensive collaboration with other nations. Several European nations, such as the UK, Germany, France, and the Netherlands, have additionally demonstrated close cooperation. China, Japan, and South Korea play important roles in sustaining international collaboration. The high centrality of the USA and UK indicates their roles as key contributors to ISS research. **Figure 2B** presents the collaboration patterns among major institutions. Notable cooperative clusters included the Children’s Hospital of Philadelphia and University of Pennsylvania (pink cluster); Leiden University and University of California, Los Angeles (blue cluster); Oregon Health & Science University and University of Gothenburg (red cluster); and Eli Lilly and Company (Indianapolis, IN, USA) with the University Children’s Hospital, Zürich (green cluster). As per **Table 2**, institutions, such as the University Children’s Hospital Zürich, University of Tübingen, and Hospital Universitario La Paz demonstrated high centrality, highlighting their influence in advancing ISS research.

**Table 1:** Top five nations by publication volume in ISS research.

Rank	Nations by publication count	Count (%)	Nations by centrality	Centrality
1	United States of America	465 (20.97)	United States of America	0.38
2	Germany	179 (8.07)	United Kingdom	0.23
3	United Kingdom	156 (7.04)	Germany	0.13
4	China	144 (6.50)	Sweden	0.12
5	Italy	140 (6.31)	France	0.1

**Table 2:** Top five institutions by publication volume and centrality in ISS research.

Rank	Institutions by publication count	Count (%)	Institutions by centrality	Centrality
1	Leiden University	60 (1.91)	Children’s Hospital, University of Zürich	0.16
2	University of Gothenburg	48 (1.52)	University of Tübingen	0.1
3	Oregon Health & Science University	33 (1.05)	Universitario La Paz	0.1
4	Children’s Hospital, University of Zürich	32 (1.02)	Centre Hospitalier Universitaire Sainte-Justine	0.09
5	University of Paris 05	29 (0.92)	National Institutes of Health (NIH)	0.09

**Figure 2:**
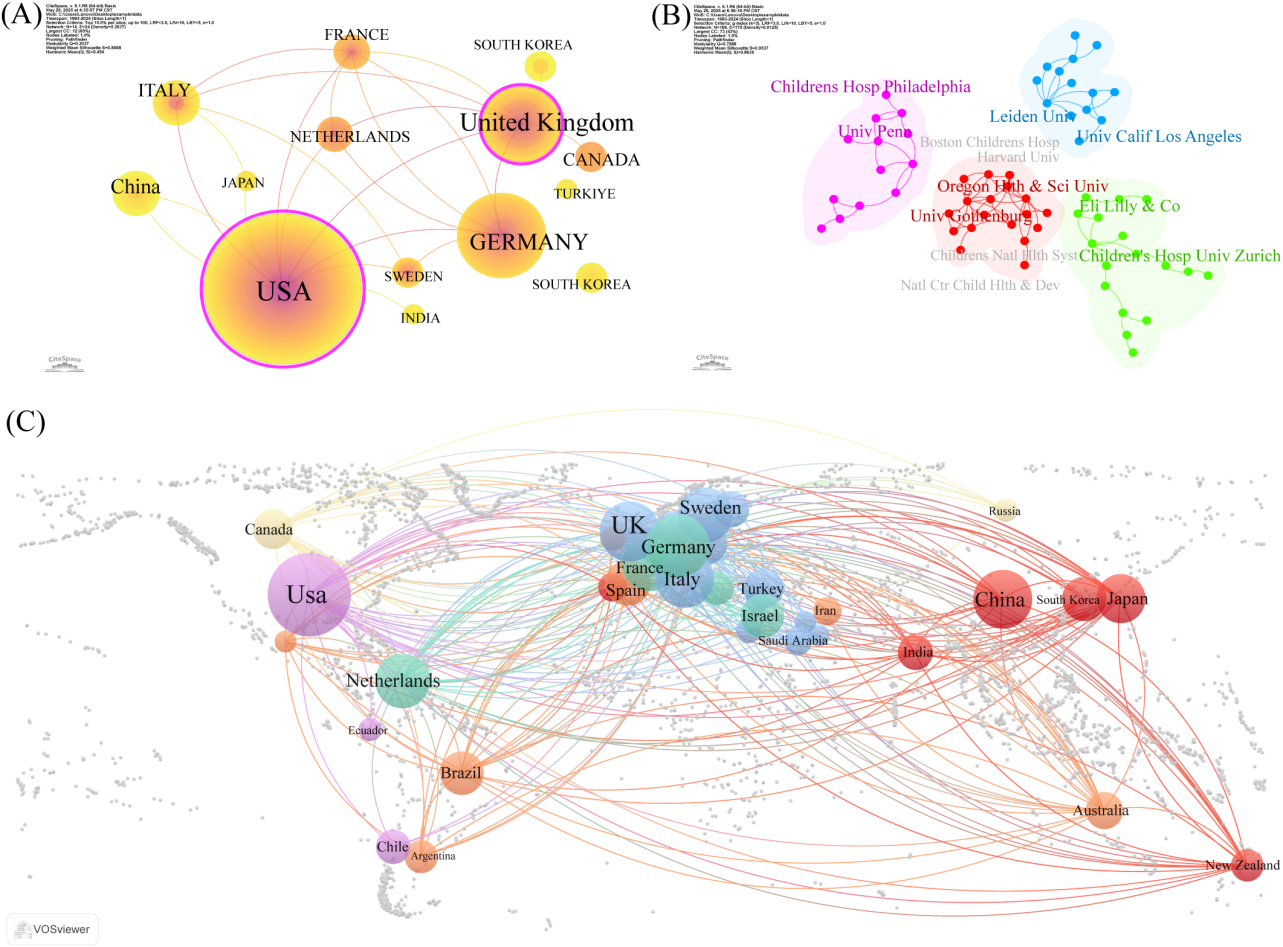
Co-authorship and collaboration analysis of nations and institutions in ISS research. **(A)** National level collaboration networks generated by CiteSpace 6.1.R6 (Chaomei Chen, Drexel University, Philadelphia, PA, USA) is depicted. Each node represents a country, node size reflects publication volume, and purple rings indicate high betweenness centrality. Thicker connecting lines denote stronger collaboration strength between nations. The United States of America (USA), United Kingdom (UK), and Germany occupy central positions in the global collaboration network. **(B)** Institutional co-authorship clusters have been identified by CiteSpace. Different colours represent distinct collaborative clusters. Notable collaborations are observed between the Children’s Hospital of Philadelphia and University of Pennsylvania (pink); Leiden University and University of California, Los Angeles (UCLA) (blue); Oregon Health & Science University and the University of Gothenburg (red); Eli Lilly and Company (Indianapolis, IN, USA) and the University Children’s Hospital Zürich (green). **(C)** Global cooperation map of institutions based on geographic distribution is shown. Circle size reflects the number of publications, connecting lines indicate inter-institutional collaboration, and colours denote regional clusters. The USA, UK, and Germany show extensive international partnerships, highlighting their leadership in ISS research.

### 3.3 Author and co-citation network analysis

**Figure 3:**
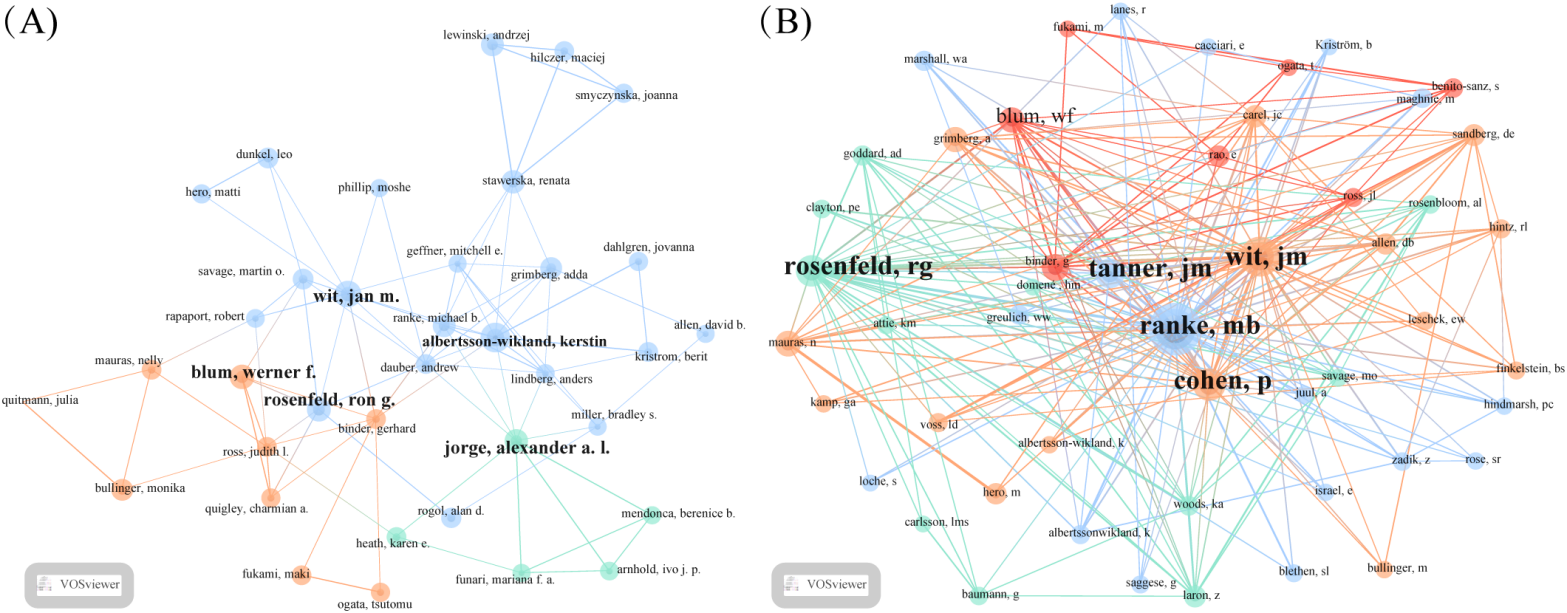
Co-authorship and co-citation analysis of key authors in ISS research. **(A)** Author co-authorship network generated using the VOSviewer (Leiden University, Centre for Science and Technology Studies, Leiden, Netherlands) is depicted. Each node represents an author, node size reflects the publication volume, and line thickness indicates the frequency of collaboration. Wit, Rosenfeld, Blum, Albertsson-Wikland, and Jorge are identified as central contributors with strong collaborative ties. **(B)** Author co-citation network is shown. Node size reflects the total citation count, and edges indicate co-citation strength. Larger nodes, such as Rosenfeld, Tanner, Wit, Ranke, and Cohen reflect foundational influence in ISS-related research.

**Table 3** presents the five most prolific ISS researchers and their affiliations: Wit (Leiden University), Albertsson-Wikland (University of Gothenburg), Rosenfeld (Oregon Health & Science University), Blum (Eli Lilly and Company), and Jorge (University of São Paulo). Collaborative networks (**Figure 3A**) reveal these authors as central hubs, reflecting their high productivity and influential roles. Notably, Wit, Blum, Rosenfeld, Albertsson-Wikland, and Jorge emerge as central nodes primarily due to their high publication output, underscoring their prominence in ISS research. Wit serves as a bridge across multiple research teams, highlighting his influential academic and integrative role within the ISS research community. Collaborative work between Wit and Rosenfeld has primarily focused on the genetic mechanisms underlying ISS, GH–IGF-related signalling pathways, and the evaluation and standardisation of GH therapy for ISS (20–23). Wit’s collaboration with Albertsson-Wikland centers on Turner syndrome management and bone age assessment tool application (24, 25). Albertsson-Wikland and Rosenfeld have co-developed predictive models for adult height outcomes in patients with GH-treated ISS (26). Rosenfeld and Blum have contributed to large-scale, prospective, international cohort studies on rhGH therapy for ISS, generating valuable clinical data that support current treatment paradigms (27). **Table 4** presents the top five most cited authors in ISS research. The co-citation network analysis (**Figure 3B**) further revealed influential scholars who played key roles in shaping the ISS research landscape. Rosenfeld, Tanner, Wit, Ranke, and Cohen had the largest node sizes, indicating both frequent citation and foundational contributions. Rosenfeld pioneered studies on the GH–IGF axis (28, 29), Tanner developed widely used growth standards (24, 30), and Wit, Cohen, and Ranke played leading roles in establishing diagnostic and therapeutic consensus guidelines (22).

**Table 3:** Top five authors by publication volume in ISS research.

Rank	Author	Publications	Nation	Institution
1	Wit	58	Netherlands	Leiden University
2	Albertsson-Wikland	42	Sweden	University of Gothenburg
3	Rosenfeld	29	United States of America	Oregon Health & Science University
4	Blum	28	Germany	University of Giessen
5	Jorge	21	Brazil	University of São Paulo

**Table 4:** Top five most cited authors in ISS research.

Rank	Cited author	Citation frequency	Nation	Institution
1	Ranke	441	Germany	University of Tübingen
2	Wit	422	Netherlands	Leiden University
3	Cohen	316	United States of America	Rockefeller University
4	Rosenfeld	300	United States of America	Oregon Health & Science University
5	Tanner	283	United Kingdom	University of London, Institute of Child Health

### 3.4 Journal and co-cited journal analysis

**Figure 4:**
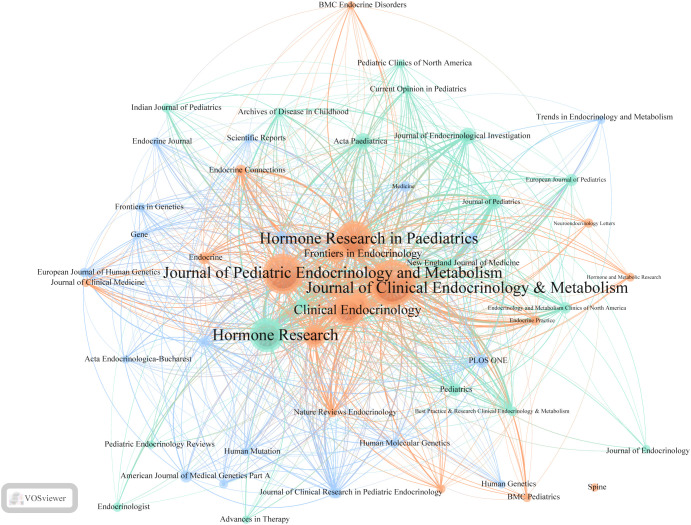
Journal co-citation network in ISS research. This visualisation depicts co-citation relationships among journals publishing ISS-related studies. Each node represents a journal, with node size indicating the citation count. Links between nodes reflect the co-citation frequency, with line thickness reflecting the strength of these connections. Different colours represent distinct clusters of journals sharing similar citation patterns. Central nodes, such as the *Journal of Clinical Endocrinology & Metabolism*, *Journal of Paediatric Endocrinology and Metabolism*, *Hormone Research in Paediatrics*, and *Clinical Endocrinology*, highlight their pivotal role in ISS research. Peripheral yet densely connected journals, including *Frontiers in Endocrinology*, *Endocrine Connections*, and *Human Genetics* emphasise the multidisciplinary nature and expanding scope of ISS-related studies.

**Table 5** lists the top five journals by publication volume in the ISS field. The *Journal of Clinical Endocrinology & Metabolism* (*JCEM*; impact factor [IF]: 5.0) recorded the highest number of publications, with 145 articles. This was followed by the *Journal of Paediatric Endocrinology and Metabolism* (*JPEM*; IF: 1.4), which published 114 articles, and *Hormone Research in Paediatrics* (*HRP*; IF: 2.6), with 98 articles. **Table 6** presents the most frequently cited journals, with *JCEM* (IF: 5.0) ranked first again, followed by *HRP* and the *Journal of Pediatrics* (*JP*; IF: 3.9). **Figure 4** illustrates the journal co-citation and publication networks within the ISS field. Each node in the network represents an academic journal, with node size corresponding to the publication volume. Edges between nodes indicate citation relationships, and edge thickness reflects the co-citation frequency. Different colours denote distinct clusters of journals sharing similar research themes or disciplinary affiliations. The *JCEM*, *JPEM*, *HRP*, and *Clinical Endocrinology* emerged as central nodes, exhibiting both high publication output and citation frequencies. These findings emphasise their prominent academic status and influence in the ISS research domain.

**Table 5:** Top 5 journals by publication volume in the field of ISS.

Rank	Journal	Publications (%)	Impact Factor (IF, Journal Citation Reports [JCR] 2025)	JCR quartile
1	*Journal of Clinical Endocrinology & Metabolism*	145 (9.50)	5	Q1
2	*Journal of Pediatric Endocrinology & Metabolism*	114 (7.47)	1.4	Q3
3	*Hormone Research in Paediatrics*	98 (6.48)	2.6	Q1
4	*Clinical Endocrinology*	78 (5.1)	3	Q2
5	*European Journal of Endocrinology*	49 (3.21)	5.3	Q1

Q1–Q4: JCR quartile ranking, with Q1 indicating the top 25% of journals in the category.

**Table 6:** Top five most cited journals in the field of ISS.

Rank	Cited journal	Citations	Impact Factor (IF, Journal Citation Reports [JCR] 2025)	JCR quartile
1	*Journal of Clinical Endocrinology & Metabolism*	1, 353	5	Q1
2	*Hormone Research in Paediatrics*	883	2.6	Q1
3	*Journal of Pediatrics*	881	3.9	Q1
4	*Clinical Endocrinology*	816	3	Q2
5	*European Journal of Endocrinology*	629	5.3	Q1

Q1–Q4: JCR quartile ranking, with Q1 indicating the top 25% of journals in the category.

In addition to these core journals, *Hormone Research*, *Frontiers in Endocrinology*, *Endocrine Connections*, and *Paediatric Endocrinology Reviews* additionally occupied key positions within the network. These journals contributed to the formation of multiple high-density citation clusters, suggesting their prominent role in shaping subfields of ISS research and facilitating knowledge dissemination. Cluster analysis revealed several thematically distinct journal groupings. The red cluster primarily comprises journals in clinical and paediatric endocrinology, focusing on disease mechanisms, diagnostic approaches, and rhGH therapy. The green cluster, represented by *Human Mutation* and *Human Genetics*, highlights the genetic and molecular mechanisms underlying ISS. The blue cluster includes general paediatric journals, such as the *JP* and the *European Journal of Pediatrics*. The yellow and cyan clusters consist of interdisciplinary journals, including *PLOS ONE* and *Scientific Reports*, reflecting the growing trend of cross-disciplinary collaboration and integration of ISS research within broader biomedical contexts. Thus, a stable and well-structured knowledge dissemination network centred on several high-impact journals has been established for ISS across endocrinology, genetics, and paediatrics. Identifying these high-output, high-impact journals enable researchers to better understand the field’s developmental trajectory, select appropriate publication avenues, and remain informed of emerging research trends.

### 3.5 Keyword analysis

**Figure 5:**
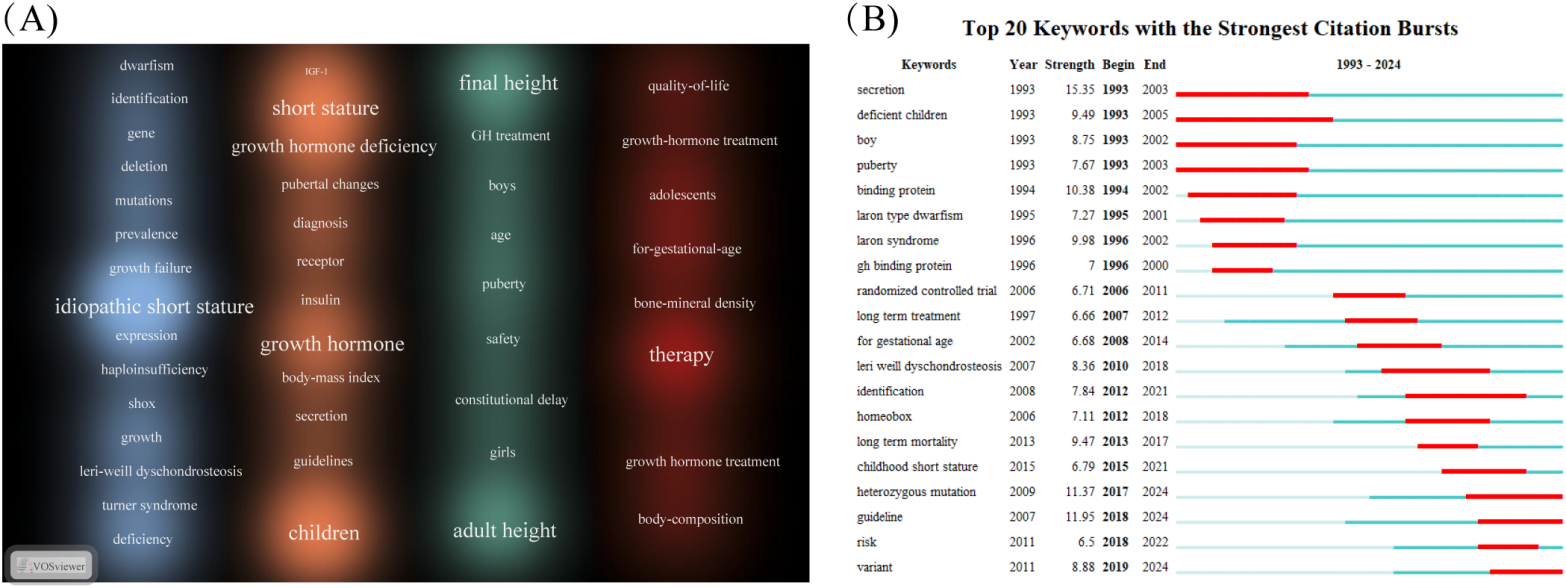
Keyword visualisation in the field of ISS. **(A)** Keyword density map generated using the VOSviewer (Leiden University, Centre for Science and Technology Studies, Leiden, Netherlands) is depicted. Font size and colour intensity reflect term frequency; with warmer colours, such as red and orange, indicating higher citation density. Frequently occurring terms, including “idiopathic short stature”, “growth hormone”, “children”, “final height”, and “therapy” highlight central research themes in this field, encompassing clinical diagnosis, treatment strategies, molecular mechanisms, and outcome evaluations. **(B)** Top 20 keywords with the strongest citation bursts from 1993 to 2024, identified using CiteSpace (Chaomei Chen, Drexel University, Philadelphia, PA, USA). Red bars represent time periods of rapid increases in citation frequency, signalling emerging research trends. Early bursts focused on endocrine function and growth hormone secretion, including terms such as, “secretion”, “binding protein”, “puberty”. By contrast, more recent bursts involved terms such as, “heterozygous mutation”, “guideline”, “risk”, and “variant”, reflecting increasing attention to genetic etiologies and individualised treatment approaches.

To elucidate research hotspots and thematic structures within the ISS field, a keyword density map was generated using VOSviewer (**Figure 5A**). Font size indicated the frequency of each keyword’s occurrence. Keywords were broadly categorised into four clusters, highlighting the multidimensional scope of ISS research. The blue cluster emphasised the molecular basis and genetic variations associated with ISS, featuring terms, such as “idiopathic short stature”, “*SHOX*”, “haploinsufficiency”, “mutation”, “gene”, “deletion”, and “Léri–Weill dyschondrosteosis”. These keywords emphasised the importance of genetic factors, particularly gene deletions and mutations, in the pathogenesis of ISS, which constituted a fundamental area of etiological research. The orange cluster pertains to clinical diagnosis and treatment, featuring keywords, such as “growth hormone”, “growth hormone deficiency”, “IGF-1”, “receptor”, “diagnosis”, “guidelines”, “secretion”, and “insulin”. This cluster centred on the GH–IGF-1 axis, receptor abnormalities, and clinical guidelines, representing core research domains in ISS pathophysiology and therapeutic decision-making. The green cluster includes keywords, such as “final height”, “adult height”, “GH treatment”, “safety”, “puberty”, and “constitutional delay”, indicating an increasing focus on the efficacy and long-term safety of rhGH therapy in patients with ISS. The red cluster highlights the impact of rhGH treatment on quality of life and the necessity for personalised therapeutic approaches tailored to different subpopulations. Keywords, such as “therapy”, “quality of life”, “growth hormone treatment”, “small for gestational age”, “body composition”, “adolescents”, and “bone mineral density” indicate a paradigm shift from exclusively improving height outcomes to addressing patient heterogeneity and broader health-related quality of life considerations.

Burst keyword analysis using CiteSpace (**Figure 5B**) was conducted to examine emerging trends between 1993 and 2024. Red bars have been used to indicate periods of high citation intensity. Longer durations reflected sustained scholarly attention, whereas higher burst strengths indicate greater academic impact. During the early years (1993–2003), research focused on foundational endocrinological topics, including “secretion”, “deficient children”, “boy”, and “puberty”. These studies emphasised GH secretion dynamics, clinical phenotypes, and the role of puberty in growth, laying the groundwork for subsequent classification and pathophysiological studies in ISS. From 2000 onward, research gradually shifted toward clinical investigations, as reflected by keywords, such as “GH binding protein”, “randomised controlled trial”, and “long-term treatment”. This trend indicated a transition from observational studies to evidence-based therapeutic evaluations, particularly concerning the long-term efficacy and standardisation of rhGH therapy. As of 2015, ISS research has increasingly focused on genetic mechanisms and the development of individualised therapeutic strategies. Emerging keywords, such as “heterozygous mutation”, “guideline”, “variant”, and “risk” reveal current priorities in identifying pathogenic gene variants, evaluating treatment-associated risks, and refining clinical protocols. This trend reflects a deeper exploration of genotype-driven aetiology and advances in standardised precision medicine approaches for the clinical management of ISS.

### 3.6 Reference analysis

**Table 7** ranks the 10 most-cited core publications in ISS research, based on citation frequency analysis. The two most frequently cited publications, each with 82 citations, were the consensus statement by Cohen et al. (2008) published in the *JCEM* (1), and GH treatment guidelines by Grimberg et al. (2016) published in *HRP* (9). These influential publications comprehensively outlined the definition, diagnostic criteria, and treatment recommendations for ISS, providing standardised clinical guidance on a global scale. Consequently, they have been widely cited and have had a significant academic impact. The third most cited study, by Goddard et al. (1995) (31), published in the *New England Journal of Medicine*, was the first to report GH receptor gene mutations in patients with ISS. This finding revealed that GH insensitivity may contribute to the aetiology of ISS, thereby broadening our understanding of its genetic basis. Subsequent influential studies included those by Wit et al. (2005) (32), Leschek et al. (2004) (33), and Finkelstein et al. (2002) (34). Wit et al. evaluated the impact of varying rhGH dosages on final height. Leschek et al. conducted randomised, double-blind, placebo-controlled trials. Finkelstein et al. performed a comprehensive meta-analysis of GH treatment in ISS. These studies have considerably strengthened the evidence supporting rhGH therapy in patients with Turner syndrome. Additionally, Attie et al. (1995) (35) utilised a large-scale national cohort study to identify GH insensitivity phenotypes in a subset of children with ISS, providing epidemiological support for recognising atypical responders. In the French *Safety and Appropriateness of Growth hormone treatments in Europe* (SAGhE) study, Carel et al. (2012) (36) raised concerns regarding a potential association between rhGH treatment and increased long-term mortality risk, prompting critical discussions concerning the long-term safety of GH therapy. Wit et al. (2008) (37) systematically reviewed the definition, epidemiology, and diagnostic approach to ISS, establishing a theoretical framework for its clinical classification and management. Collectively, these frequently cited references constitute the foundational knowledge base for ISS research. They address essential domains, including disease definition and diagnosis, genetic aetiology, treatment efficacy, long-term safety, and formulation of clinical guidelines. These studies continue to inform clinical decision-making and shape future research directions in the field.

**Table 7:** Top 10 most frequently cited references in ISS research.

Rank	Frequency	References	Source	Author and year of publication
1	82	Consensus Statement on the Diagnosis and Treatment of Children With Idiopathic Short Stature: A Summary of the Growth Hormone Research Society, Lawson Wilkins Pediatric Endocrine Society, and European Society for Paediatric Endocrinology Workshop	*Journal of Clinical Endocrinology & Metabolism*	Cohen et al., 2008 (1)
2	82	Guidelines for Growth Hormone and Insulin-Like Growth Factor-I Treatment in Children and Adolescents: Growth Hormone Deficiency, Idiopathic Short Stature, and Primary Insulin-Like Growth Factor-I Deficiency	*Hormone Research in Paediatrics*	Grimberg et al., 2016 (9)
3	49	Mutations of the Growth Hormone Receptor in Children With Idiopathic Short Stature. The Growth Hormone Insensitivity Study Group	*New England Journal of Medicine*	Goddard et al., 1995 (31)
4	46	Diagnosis, Genetics, and Therapy of Short Stature in Children: A Growth Hormone Research Society International Perspective	*Hormone research in paediatrics*	Collett-Solberg et al., 2019 (22)
5	40	Growth Hormone (GH) Treatment to Final Height in Children With Idiopathic Short Stature: Evidence for a Dose Effect	*Journal of Pediatrics*	Wit et al., 2005 (32)
6	39	Effect of Growth Hormone Treatment on Adult Height in Peripubertal Children With Idiopathic Short Stature: A Randomized, Double-Blind, Placebo-Controlled Trial	*Journal of Clinical Endocrinology & Metabolism*	Leschek et al., 2004 (33)
7	38	Evidence for Partial Growth Hormone Insensitivity Among Patients With Idiopathic Short Stature. The National Cooperative Growth Study	*Journal of Pediatrics*	Attie et al., 1995 (35)
8	37	Long-term Mortality After Recombinant Growth Hormone Treatment for Isolated Growth Hormone Deficiency or Childhood Short Stature: Preliminary Report of the French SAGhE Study	*Journal of Clinical Endocrinology & Metabolism*	Carel et al., 2012 (36)
9	36	Idiopathic Short Stature: Definition, Epidemiology, and Diagnostic Evaluation	*Growth Hormone & IGF Research*	Wit et al., 2008 (37)
10	32	Effect of Growth Hormone Therapy on Height in Children With Idiopathic Short Stature: A Meta-Analysis	*Archives of Pediatrics & Adolescent Medicine*	Finkelstein et al., 2002 (34)

### 3.7 Clinical progress analysis

**Figure 6:**
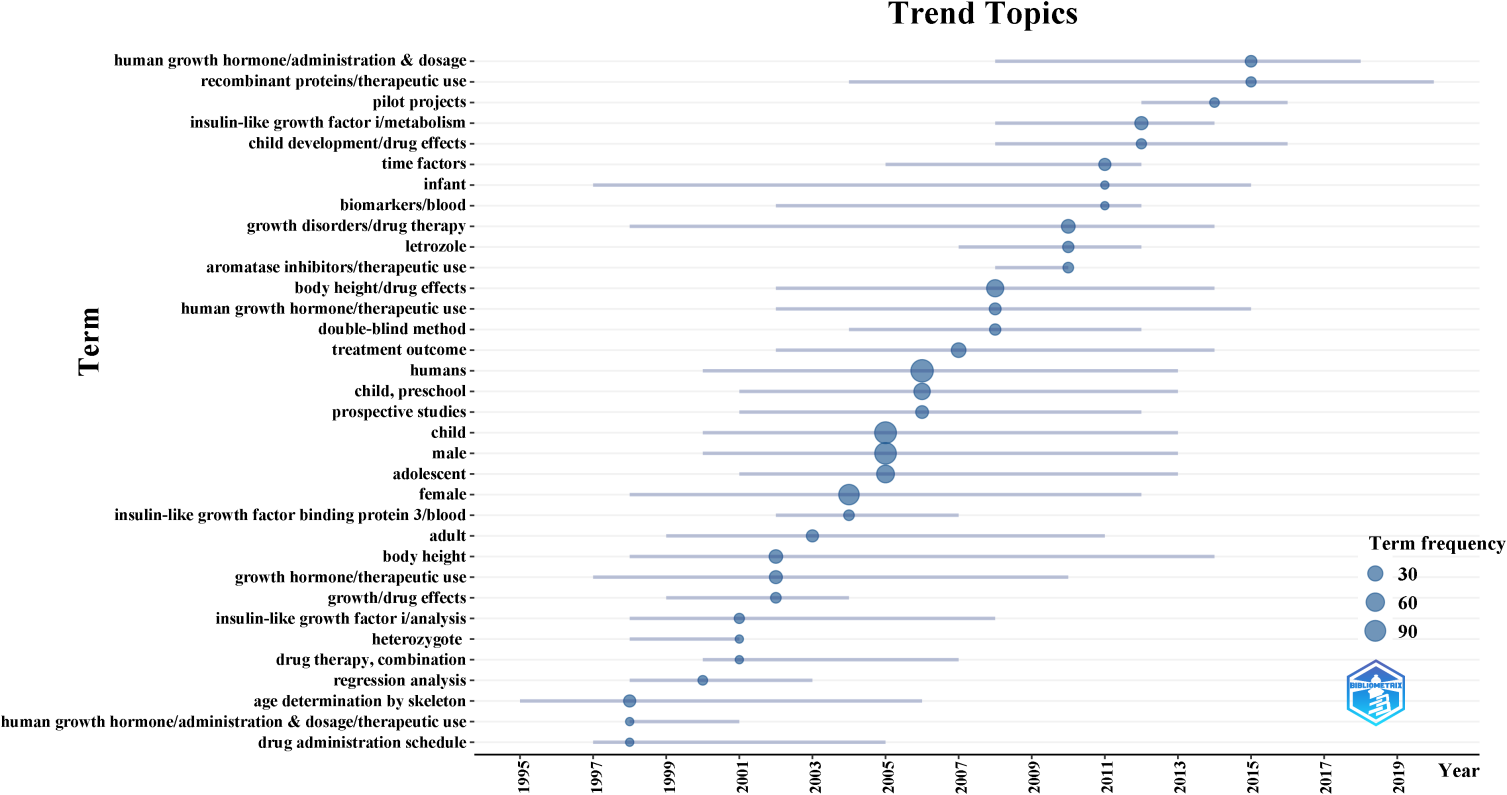
Evolution of clinical research focus in ISS from 1993 to 2024. This timeline, generated using the R package *bibliometrix* based on 113 PubMed-indexed clinical trials, visualizes key MeSH terms and their periods of prominence. Early research (1990s–2000s) focused on diagnostic and therapeutic fundamentals, including “age determination by skeleton, “ “human growth hormone/administration & dosage, “ and “insulin-like growth factor I/analysis.” In contrast, recent studies emphasize “drug administration schedule, “ “regression analysis, “ and “personalized therapy, “ reflecting a shift toward optimized, individualized treatment strategies.

A total of 113 clinical trials were retrieved from the PubMed database. As shown in **Figure 6**, we analyzed the progress of clinical trials in the field of ISS. The clinical research on ISS has evolved significantly over the past few decades, with an increasing focus on therapeutic advancements following initial foundational studies. In the late 1990s, research was primarily centered on understanding growth mechanisms, as reflected in studies on “age determination by skeleton” (1995–2006) and “human growth hormone/administration & dosage” (1998–2001). These early investigations were crucial for diagnosing growth disorders and establishing the role of human growth hormone in the treatment of ISS. During this period, determining the optimal dosage and administration schedule of human growth hormone (HGH) was a key area of exploration, as researchers sought to improve treatment efficacy for children with short stature. Additionally, the recognition of insulin-like growth factor I (IGF-I) in growth regulation became more prominent, with studies focusing on “insulin-like growth factor I/analysis” (1998–2008), highlighting its potential as both a biomarker and therapeutic target. By the early 2000s, attention shifted toward refining treatment protocols, with terms such as “drug administration schedule” (1997–2005) and “regression analysis” (1998–2003) indicating a growing interest in optimizing HGH therapy. The application of statistical methods, such as regression analysis, became increasingly important for assessing treatment outcomes and understanding the long-term effects of HGH on growth trajectories. This period marked a transition from a focus on basic hormonal treatment to more sophisticated data-driven approaches that considered individualized patient responses. In recent years, the field has expanded its scope to include more personalized treatment strategies, exploring combination therapies and refined regimens tailored to specific patient needs. This shift reflects a deeper understanding of the multifactorial nature of ISS and the recognition that treatment must go beyond standard HGH administration. Clinical trials now examine the interplay between growth hormones, IGF-I, and other molecular factors, alongside statistical models that evaluate long-term therapeutic impact. This progression in clinical research highlights the increasing complexity of ISS treatment, emphasizing the need for continuous refinement of therapeutic protocols to optimize outcomes for children with short stature.

## 4 Discussion

In this study, we systematically mapped the developmental trajectory and current status of ISS research from 1993 to 2024 through a multidimensional bibliometric analysis, which included annual publication trends, national and institutional collaboration networks, influential authors and journals, keyword evolution, and frequently cited references. Annual publications have steadily increased over the past three decades, particularly since 2010. Despite a slight decline since 2021, the overall publication volume remained high, indicating that ISS has become a prominent research topic in paediatric endocrinology and has received increasing global attention. This sustained growth highlights the persistent clinical challenge posed by ISS and the ongoing scientific efforts to unravel its complexities.

Collaboration network analysis revealed that nations, such as the USA, Germany, and the UK held central positions in ISS research, with the USA exhibiting high centrality, highlighting its academic leadership. Research contributions from Asian nations, including China, Japan, and South Korea, have increased in recent years, promoting diversity and international collaboration. The top three most productive institutions were Leiden University (Netherlands), University of Gothenburg (Sweden), and Oregon Health & Science University (USA). By contrast, institutions, such as the University Children’s Hospital Zürich, University of Tübingen, and Hospital Universitario La Paz demonstrated high centrality, reflecting their pivotal roles within the ISS research network. Author collaboration and co-citation analyses reveal the emergence of a relatively stable core academic community in ISS research. These highly productive and frequently cited scholars have driven both fundamental research advances and therapeutic innovation, while directly influencing clinical practice through consensus guidelines and standardized protocols. This dual impact reflects a maturation phase in ISS research, characterized by robust academic collaboration and cumulative knowledge integration.

Keyword-density and burst analyses reveal the dynamic evolution of ISS research themes. Early work focused primarily on GH secretory mechanisms and clinical phenotypes, laying the groundwork for disease understanding and initial therapeutic approaches. Subsequently, attention shifted to evaluating the efficacy and long-term safety of rhGH, thereby facilitating its widespread clinical adoption. In recent years, research has progressively expanded to encompass genetic diagnosis and putative genetic mechanisms, aligning with the broader shift in medicine towards precision and individualized treatment. Human height exemplifies a polygenic trait where additive effects of multiple variants create continuous, genetically determined phenotypic variation (38). Within ISS research, molecular genetics has thus assumed increasing prominence. Pathogenic variants in genes such as FGFR3, SHOX, and GHR have been associated with ISS (39–41). Genetic testing now enables clinicians not only to establish an aetiological diagnosis but also to identify comorbidities and refine treatment plans (5, 42). Databases (e.g., GWAS Catalog, Deciphering Developmental Disorders) continually expand ISS-related variant collections, while clinical data reciprocally enrich these resources, creating a translational research-practice cycle. For example, pathogenic FGFR3 variants induce constitutively upregulated signaling that suppresses proliferation and differentiation in growth-plate chondrocytes, leading to short stature; genetically confirmed cases demonstrate indications for vosoritide therapy (39). Conversely, GHR mutations or STAT5B deficiency predict a suboptimal response to rhGH, necessitating therapeutic reassessment. These findings support expanding genetic testing in clinical care to enable precision and individualized interventions. However, implementation challenges persist. Rigorous cost-effectiveness analyses are needed to justify diagnostic and therapeutic expenditures, while regional and familial disparities in affordability limit access. Translating genetic discoveries into clinical decisions (e.g., GH regimen selection and dose adjustment) will require further large-scale studies. Notably, the recent emergence of “Guideline” in burst analysis reflects influential international consensus statements (9, 22) that standardize etiological classification, treatment pathways, and monitoring protocols, advancing global practice harmonization.

The consensus guidelines published by Cohen et al. (2008) (1) and Grimberg et al. (2016) (9) have been extensively cited and offer standardised definitions, diagnostic pathways, and treatment strategies, providing a globally recognised framework for clinical management. In 1995, Goddard et al. (31) advanced the genetic understanding of ISS by identifying GH receptor gene mutations associated with GH insensitivity. Additional frequently cited studies have addressed GH dosing, long-term safety, and adverse events associated with rhGH therapy (32–34), reflecting ongoing efforts to balance therapeutic efficacy and patient safety. Despite these advances, the management of ISS remains challenging. Currently, rhGH is the most widely used treatment for ISS. Since its approval by the USA FDA in 2003, rhGH has been adopted in countries including China, Australia, and New Zealand. Multiple studies have confirmed its effectiveness in improving final height in children, with outcomes showing a dose-dependent response (10, 11, 33). However, concerns regarding their long-term safety persist. Some studies have documented elevated fasting insulin levels and increased insulin resistance in patients with ISS receiving rhGH (43). The 2012 French SAGhE study identified a higher mortality risk due to bone tumours or intracranial haemorrhage in adults previously treated with rhGH in childhood, with risk correlating to dosage (36). The 2020 SAGhE study, which included 24, 232 children, found increased all-cause mortality associated with specific underlying diagnoses. However, no overall increase in mortality was observed in patients with ISS. Nevertheless, the elevated risk of cerebrovascular mortality in patients treated with rhGH is consistent with previous findings (12). Additionally, the clinical applications of long-acting PEGylated rhGH are continuously expanding; however, its long-term efficacy and safety require further investigation. In 2023, Wit emphasised the need for strengthened post-marketing surveillance of PEGylated rhGH and advocated a cautious approach that balances therapeutic innovation with safety monitoring (44).

In addition to rhGH therapy, GnRH analogues (GnRHas) and aromatase inhibitors have frequently been used in combination therapies to delay skeletal maturation and extend the period of linear growth. During puberty, accelerated bone age in patients with ISS can limit growth potential, particularly in those receiving rhGH monotherapy. GnRHa suppresses the hypothalamic–pituitary–gonadal axis, delaying puberty. GnRHa alone has demonstrated limited effectiveness in increasing the final height (45). However, when administered early in puberty alongside rhGH therapy, this combination is associated with improved adult height outcomes (46, 47). A temporary reduction in growth velocity may occur; however, long-term outcomes for adult height are favourable. Clinical studies have shown that GnRHas are generally well tolerated, with minimal adverse effects and normal resumption of pubertal development following treatment cessation (48). Aromatase inhibitors, typically prescribed for boys with advanced bone age, inhibit the conversion of androgens to estrogens, thereby delaying epiphyseal fusion and supporting continued linear growth. The efficacy of aromatase inhibitors has been confirmed in multiple studies, particularly when used in combination with rhGH (49–51). Nevertheless, clinicians should remain vigilant regarding potential adverse effects, including hyperandrogenism, neuropsychological symptoms such as drowsiness and memory loss, low high-density lipoprotein levels, arthralgia, hyperinsulinemia, and hyperuricemia. Most adverse effects resolve within 3 months of discontinuing treatment. The impact of aromatase inhibitors on reproductive function in children remains unclear and requires further investigation (52).

Clinical progress analysis indicate that recent ISS research has increasingly shifted from a single therapeutic strategy toward more individualized and precision-based interventions, exploring combination therapies and refined approaches tailored to specific patient characteristics. Currently, rhGH remains the standard treatment for ISS; however, in recent years, combination regimens involving rhGH with GnRHa or aromatase inhibitors have gained growing attention in pubertal patients. These strategies aim to extend the growth period and improve adult height outcomes. Nevertheless, the long-term safety and efficacy of such combination therapies require further investigation and validation. Meanwhile, with the growing clinical adoption of genomic medicine, personalized treatment strategies based on genetic characteristics are showing growing potential. For example, Mecasermin may be considered for patients with GHR mutations and low IGF-1 levels, whereas Vosoritide may benefit children with FGFR3 mutations. This emerging trend reflects a paradigm shift in ISS treatment—from the traditional “one-size-fits-all” rhGH therapy to a more stratified and precise therapeutic model guided by genetic and biomarker profiles.

Overall, despite substantial advances in ISS research, current studies remain largely focused on genetic mechanisms and growth outcomes, with comparatively limited attention to children’s psychosocial health, cognitive development, and long-term metabolic outcomes (53, 54). Furthermore, the clinical evidence base predominantly derives from high-income countries, potentially limiting external validity for resource-limited settings. Based on developments over the past 32 years, ISS research is likely to diversify further over the next 5–10 years. Beyond existing genetic paradigms, future research is expected to prioritize psychosocial and cognitive outcomes alongside metabolic profiling. At the same time, systematic functional studies of clinically identified pathogenic gene variants will be essential to deepen our understanding of ISS pathogenesis. The integration of multi-omics approaches—including transcriptomics, proteomics, and metabolomics—holds great promise for elucidating the underlying mechanisms of ISS, thereby advancing precision diagnostics and enabling stratified treatment strategies. As clinical indications for long-acting growth hormone continue to expand, related clinical trials in pediatric ISS populations are expected to increase. Meanwhile, more emphasis should be placed on real-world implementation studies of newly developed clinical guidelines, evaluating their actual adoption, implementation quality, and cost-effectiveness to determine their clinical feasibility and health-economic value. Notably, advancements in artificial intelligence have the potential to revolutionize care delivery through automated bone-age imaging analysis, multimodal treatment optimization, and predictive modeling of adult height—collectively enhancing diagnostic accuracy and therapeutic decision-making.

This study has several limitations. First, although the literature search covered publications up to December 2024, data extraction was completed at an earlier date, which may have excluded the most recent studies and thus limited the assessment of current research trends. Second, bibliometric analyses rely primarily on quantitative indicators, such as publication and citation counts, without sufficiently accounting for study quality, methodological rigor, or scientific value; as a result, the academic impact of frequently cited articles may be overstated. Third, only English-language publications were included, which may have introduced language bias and underestimated contributions from non-English-speaking countries. Finally, the WoSCC was chosen as the primary database for bibliometric analysis due to its broad coverage and reliability, with PubMed additionally consulted for clinical research content; however, relevant studies indexed exclusively in other databases, such as Scopus or Embase, may still have been missed. This database selection ensured consistency and reproducibility but inevitably limited the comprehensiveness of the included literature.

In conclusion, A comprehensive bibliometric analysis mapped the literature on ISS from 1993 to 2024, presenting a systematic overview of the field’s developmental trajectory and current research priorities. Nations, institutions, and researchers based in North America and Europe occupy central positions in the progression of ISS-related work. Research emphasis included the identification of pathogenic genes, assessment of treatment-related risks, and standardisation of diagnostic and therapeutic protocols. Overall, the findings offer a structured reference for outlining knowledge frameworks and academic contributions. New investigators can access essential insights by locating foundational literature, recognising influential contributors, and identifying appropriate journals to inform their submission strategies and future research directions.

## Data Availability

The original contributions presented in the study are included in the article/supplementary material. Further inquiries can be directed to the corresponding authors.
